# Bacterial infections and antibiotic susceptibility in children with sickle-cell anemia on malaria chemoprevention using Dihydroartemisinin-piperaquine (DP) or Sulfadoxine-pyrimethamine (SP) in Uganda

**DOI:** 10.1371/journal.pone.0340838

**Published:** 2026-10-15

**Authors:** Winnie Nambatya, Moses Ocan, Grace Ndeezi, Dennis Kalibbala, Ritah Namusoosa, Freddie Bwanga, Thandile Nkosi-Gondwe, Joanita Kirikumwino, Alisen Ayitewala, Isaac Ssewanyana, John Ssenkusu, Robert Opoka, Feiko O. ter Kuile, Kamija S. Phiri, Bjarne Robberstad, Richard Idro

**Affiliations:** 1 Department of Pharmacy, College of Health Sciences, Makerere University, Kampala, Uganda; 2 Department of Pharmacology and Therapeutics, College of Health Sciences, Makerere University, Kampala, Uganda; 3 Department of Paediatrics and Child Health, College of Health Sciences, Makerere University, Kampala, Uganda; 4 Global Health Uganda, Kampala, Uganda; 5 National Microbiology Reference Laboratory, National Health Laboratory and Diagnostic Services, Ministry of Health, Kampala, Uganda; 6 Department of Immunology and Molecular Biology, College of Health Sciences, Makerere University, Kampala, Uganda; 7 Training and Research Unit of Excellence, Blantyre, Malawi; 8 Department of Epidemiology and Biostatistics, Makerere University, College of Health Sciences, Kampala, Uganda; 9 Department of Clinical Sciences, Liverpool School of Tropical Medicine, Liverpool, United Kingdom; 10 School of Global and Public Health, Kamuzu University of Health Sciences, Blantyre, Malawi; 11 Department of Global Public Health and Primary Care, University of Bergen, Bergen, Norway; George Washington University School of Medicine and Health Sciences, UNITED STATES OF AMERICA

## Abstract

**Introduction:**

Over 300,000 babies are born annually with sickle cell anemia (SCA) in sub-Saharan Africa. Despite malaria chemoprevention and early penicillin prophylaxis, these children develop febrile illnesses. The effect of malaria chemoprevention regimens on the incidence of bacterial infections and antimicrobial resistance is unclear. We assessed incidence of bacterial infections and resistance patterns in children with SCA receiving weekly DP or monthly SP.

**Methods:**

This study was nested in a randomized, double-blind clinical trial comparing weekly DP with monthly SP. Participants were children aged 6 months to 15 years with SCA, followed every two months for a median of 14.7 months (IQR 11.2–18.2) and evaluated at all sick visits. Blood cultures were obtained for episodes of fever (axillary temperature >38℃) and processed using the BD BACTEC automated system. Bacterial identification was by phenotypic and genotypic methods. Whole-genome sequencing was performed using Illumina DNA Prep Kit, and antimicrobial resistance genes were characterized using AMRFinderPlus. Analyses were conducted in STATA 18.0 and limited to Ugandan participants.

**Results:**

Of 725 enrolled participants, 437 were from Uganda (221 DP; 216 SP). The mean (SD) age was 7.3 (3.8) years, and 52.4% were male. Pathogenic bacteria were rarely isolated, including *Escherichia coli* (3), *Klebsiella pneumoniae* (2), *Staphylococcus aureus* (2), *Streptococcus agalactiae*. (1), and *Enterococcus faecalis* (1). The cumulative incidence of bacterial growth over 22 months of follow-up was 2.3% (95% CI: 1.12–4.73). Children with positive cultures tended to have higher fevers and more hospitalizations. All isolates were multidrug-resistant. Multiple resistance genes were identified, including plasmid-mediated sulfonamide resistance via sul2 in both *Klebsiella pneumoniae* and *Escherichia coli.*

**Conclusion:**

Pathogenic bacteria were infrequently isolated from Ugandan children with SCA receiving malaria chemoprevention. The presence of sul2 indicates the possibility of plasmid-mediated sulfonamide resistance.

## Background

Globally, more than 300,000 children are born annually with s SCA, with approximately 75% of these births occurring in Africa [1–3]. In addition to the direct effects of SCA, such as repeated painful vaso-occlusive crises, chronic hemolysis, and anemia, children with SCA are at an increased risk of febrile illnesses, particularly from invasive bacterial infections by capsulated organisms [4] due to their immune system dysfunction. These infections contribute to premature death in children with SCA.

Bacterial infections among children with SCA persist despite advances in their prevention, such as daily oral penicillin prophylaxis and routine pneumococcal vaccination. The types of prevalent bacterial pathogens vary across studies and regions. Globally, the most commonly reported bacterial infections in children with SCA are caused by *Streptococcus pneumoniae, non-typhoidal Salmonella species,* and *Haemophilus influenzae* [5], but geographical variations exist. Studies from Nigeria and Uganda report a predominance of non-typhi *Salmonella species*, *Escherichia coli, Staphylococcus aureus,* and *Klebsiella species* [6–8], with *Streptococcus pneumoniae* and *Haemophilus influenzae* infrequently reported. A Kenyan study among children with SCA [9] reported *Streptococcus pneumoniae,* non-typhoidal *Salmonella species, Haemophilus influenzae type b, Acinetobacter species,* and *Escherichia coli*. In addition, a 2025 systematic review [5] highlighted that most samples used to assess antimicrobial resistance were obtained from urine rather than blood, despite bloodstream infections being the most common morbidity in children with SCA [10]. In Uganda, the standard of care includes daily oral penicillin prophylaxis for children under five years and routine pneumococcal vaccination [11]. However, these have a narrow bacterial spectrum; thus, the risk of bacterial infections remains high despite these interventions.

The vulnerability of individuals with SCA to bacterial infections is further nuanced by factors such as age and coexisting conditions [12–14], including malaria. While people with the heterozygous sickle cell trait experience a reduced risk of malaria, those with the homozygous SCA face increased morbidity and mortality if they contract malaria [15,16] and so are provided malaria chemoprevention. In Uganda, the standard of care for malaria chemoprevention is monthly SP. SP possesses anti-bacterial activity and may offer partial protection against bacterial infections in this population. Therefore, this study investigated the occurrence of bacterial infections in children with SCA receiving malaria chemoprevention with weekly DP or monthly SP in Uganda.

## Materials and methods

### Study design

This study was nested in a randomized, parallel-group, double-blind, placebo-controlled clinical trial (CHEMCHA) of weekly DP vs monthly SP [4].

### Study setting and target population

The study was nested in the CHEMCHA trial conducted in Uganda (Jinja Regional Referral Hospital (RRH) and Kitgum General Hospital) and Malawi from April 2021 to May 2023 among children with SCA [4,17].

### Sample size

This was a secondary analysis using blood samples collected from 437 Ugandan children enrolled in the CHEMCHA trial, a randomized, double-blind, placebo-controlled trial comparing weekly DP with monthly SP for malaria chemoprevention in children with SCA. The parent trial was designed to detect at least a 50% reduction in the incidence of clinical malaria, with 90% power and a two-sided α of 0.05. Of the 725 participants enrolled in the parent trial, 437 were from Uganda, and 288 were from Malawi [4]. Samples from the Malawi site were not stored; therefore, this study was conducted using only samples collected from Ugandan participants.

At the end of enrolment in Uganda, 221 participants were in the DP arm, and 216 were in the SP arm. The parent CHEMCHA trial reported suspected sepsis in 115 of 367 participants (31.3%) in the DP arm versus 92 of 357 participants (25.8%) in the SP arm [4], a difference of 5.6%. Detecting a difference of this magnitude with 80% power (two-sided α = 0.05) would require approximately 1,030 participants per arm using the two-proportion method [18]. With the fixed Uganda sample size of 221 and 216 participants per arm, this analysis had only approximately 25% power to detect a difference of this magnitude, and the minimum difference reliably detectable with 80% power is approximately 12.1% [18]. Therefore, the analysis in this study was descriptive and hypothesis-generating.

### Sampling procedure and selection of respondents

Participants in the main study [4] were 725 children aged 6 months to 15 years with a diagnosis of SCA on Hb electrophoresis or isoelectric focusing. For this study, only participants in Uganda were included. Children with other known chronic diseases, those who were receiving daily cotrimoxazole prophylaxis, or those who were participating in another trial were excluded.

### Data collection process

The trial procedures have been described previously [4]. Briefly, at enrolment [4], i.e., between April 17, 2021, and May 30, 2022, sociodemographic information and medical history were recorded, and a physical exam was performed [17]. Participants were randomly assigned in a 1:1 ratio to one of two malaria chemoprevention regimens using a computer-generated block randomisation sequence with randomly varying block sizes of 4, 6, or 8, stratified by site [4]. To maintain blinding, participants in each arm received one active regimen alongside a matched placebo for the alternative regimen: those assigned to the DP arm received weekly active DP and monthly placebo SP, while those assigned to the SP arm received monthly active SP and weekly placebo DP. [17]. Participants who were ill were initially resuscitated and would be enrolled when clinically stable [17]. Participant characteristics were all similar at baseline in all the groups [4].

Participants attended scheduled clinics with their caregivers every 2 months to receive their next doses of study medication and unscheduled visits if they were unwell. At each study visit, participants receiving SP were provided with two additional doses to cover the subsequent two months. Participants receiving DP were provided with doses sufficient to cover 8 weeks. These additional doses were dispensed on the last day of the preceding scheduled visit. The study provided standard-of-care clinical services for all unscheduled visits. Parents or guardians were instructed on home management of children with SCA and taking the children to the study clinic after identifying signs that require medical attention [4,17]. If the participant did not attend the scheduled follow-up visits, they were contacted by phone to remind them and visited at home if so required [4]. Participants were admitted to the hospital if they developed severe complications of SCA. Blood samples were taken for culture if children presented with an axillary temperature of 38°C or higher [4].

### Sample collection, processing, and culture

For each patient with a temperature ≥ 38 °C, 1–5 ml of venous blood was collected aseptically from the antecubital fossa and inoculated into BD BACTEC™ Peds Plus vials containing enhanced Soybean-Casein Digest broth with CO_2_ to support the growth of organisms. We transported the samples from the wards to the laboratory in ice boxes.

The inoculated vials were loaded into the BD BACTEC™ FX200 automated blood culture system (Becton Dickinson, Temse, Belgium) and incubated at 37 °C for up to 5 days (120 hours), with continuous monitoring for microbial growth. Whenever the instrument flagged a vial as positive, Gram staining and sub-culturing onto solid media were performed on the same day. If no growth was detected by the end of the 5-day incubation period, the sample was reported as negative.

### Identification of bacterial strains

Bacterial colonies were obtained through sub-culturing of positive primary blood culture samples flagged by the BD BACTEC™ system onto blood agar, chocolate agar, and MacConkey agar. After 24-hour incubation, the colonies were examined and described based on morphological characteristics, that is, shape, size, colour, odour, haemolysis patterns on blood agar and chocolate agar (alpha, beta, or gamma), colony elevation, margin appearance, surface texture, consistency when probed, opacity, and pigmentation [19].

Gram staining was done according to Claus [20]. A sterile needle was used to draw a sub-sample following aseptic techniques. A drop of blood was placed on a clean slide to make a thick smear that was then heat-fixed. The smear was flooded with the primary stain (crystal violet) for one minute, then washed off, followed by the mordant (Gram’s iodine) for one minute and then washed off again. A decolouriser (ethanol with 50% acetone) was applied until no more purple colour came off. The smear was then flooded with a counterstain (safranin) for 2 minutes and then washed. The stained slide was air-dried and observed under 100X magnification on the microscope with immersion oil [21]. The gram-positive bacteria retained the primary stain and appeared purple under the microscope, while the gram-negative bacteria took up the counterstain and appeared red.

Gram-negative organisms were identified using triple sugar iron agar, urea, Simmons citrate agar, sulfur indole motility test, and oxidase test, while gram-positive organisms were identified using catalase, coagulase, DNase, optochin sensitivity test, and bacitracin sensitivity test [22].

### Antimicrobial susceptibility testing

The antimicrobial susceptibility testing (AST) of the identified isolates to commonly used antibiotics was performed using the Kirby–Bauer disc diffusion method [23]. Colonies from overnight pure cultures were suspended in 0.85% sterile normal saline to achieve a 0.5 McFarland turbidity standard, then spread uniformly onto Mueller–Hinton agar plates using sterile cotton swabs. For Streptococcus species, Mueller–Hinton agar supplemented with 5% sheep blood was used, with incubation at 37 °C in 5% CO_2_ for 20–24 hours; all other organisms were incubated at 37 °C in ambient air for 16–18 hours. Antibiotic discs were aseptically placed on the inoculated plates within 15 minutes of inoculation, and inhibition zone diameters were measured in millimetres using a calibrated ruler. Selection of antibiotic discs and interpretation of the AST results for each organism was done following the Clinical and Laboratory Standards Institute (CLSI) guidelines, 34^th^ edition [23]. Contaminated blood cultures were defined as cultures in which the identified organism is one commonly associated with common skin flora contamination, e.g., coagulase-negative Staphylococcus, Bacillus [24,25]

### Bacterial genotyping

The bacterial genotyping was carried out at the Central Public Health Laboratories (CPHL) in Kampala, Uganda. Bacterial isolates were enriched for 24 hours in Brain Heart Infusion broth before genotyping. For Whole Genome Sequencing, genomic DNA was extracted from isolates using the QIAamp DNA Mini Kit (Qiagen), according to the manufacturer's instructions. DNA yield was quantified using the Qubit 1X dsDNA Broad Range Kit (ThermoFisher). DNA libraries were then prepared from the extracted DNA using the Illumina DNA Prep Kit (Illumina, USA) following the manufacturer's protocol. DNA Library were normalised following quantification using the Qubit™ 1X dsDNA High Sensitivity Kit (ThermoFisher). The normalised libraries were then pooled, the pooled library was quantified with the same Qubit kit, and assessed for fragment size using the Agilent High Sensitivity DNA Kit on a BioAnalyzer (Agilent). The pooled library was diluted to the appropriate loading concentration and sequenced using the Illumina MiSeq platform. Sequence reads were assembled using both reference-based and de novo approaches, and antimicrobial resistance genes or mutations were identified using AMRFinderPlus

### Data management and analysis

Data were collected and recorded at the point of contact using a combination of paper-based and electronic record forms [17]. We used the Research Electronic Data Capture (REDCap™), locally hosted. Data quality was ensured through comprehensive training of the study team, standardized questionnaire administration, data collection procedures, good clinical data management practices and good laboratory practices. Built-in range, logic, and consistency checks were incorporated into the electronic system to minimize data entry errors.

Data were analyzed using Stata version 18. Baseline demographic and clinical characteristics were summarized using means and standard deviations (SD) for continuous variables, while categorical variables were summarized as frequencies and percentages. Stratified summaries are presented by study site [4].

The main study outcome was the cumulative incidence of bacterial growth. Cumulative incidence of bacterial growth was assessed using survival analysis methods. Time to first bacterial growth was defined as the duration (in months) from enrolment to the first positive culture. Kaplan–Meier failure functions were used to estimate and plot the cumulative probability of bacterial growth. A case series description was done together with a descriptive analysis to determine frequencies, and the data were presented in tables.

### Ethics approval and consent to participate

Written consent was obtained from the parent or caregiver (and assent from children aged ≥8 years). The main study was approved by Makerere University School of Medicine Research and Ethics Committee (2020−103), Liverpool School of Tropical Medicine Research Ethics Committee (19.105), the Regional Committee for Medical and Health Research Ethics, Western Norway (30992), and the National Health Sciences Research Committee in Malawi (19/11/2442). The trial is registered at ClinicalTrials.gov, NCT04844099 [17].

## Results

### Baseline characteristics of study participants

The study enrolled 437 children with SCA in Uganda, including 248 from Jinja RRH and 189 from Kitgum General Hospital. The mean age was 7.3 (SD 3.9) years, and half, 52.4% (229/437) of the children were males. Most, 41.9% (183/437) of the caretakers had completed a secondary level of education Table 1.

**Table 1:** Social demographic and baseline clinical characteristics of the study participants.

Characteristic	DP *(n = 221)*	SP *(n = 216)*	Total *(n = 437)*
**Age, mean (SD)**	7.3 (3.9)	7.4 (3.8)	7.3 (3.9)
**Age groups**			
0- < 5 years	71 (32.6%)	70 (32.7%)	141 (32.6%)
5- < 10 years	91 (41.7%)	85 (39.7%)	176 (40.7%)
10-15 years	56 (25.7%)	59 (27.6%)	115 (26.6%)
**Sex, n (%)**			
Male	121 (54.8%)	108 (50.0%)	229 (52.4%)
Female	100 (45.2%)	108 (50.0%)	208 (47.6%)
Study site			
Jinja	127 (57.5%)	121 (56.0%)	248 (56.8%)
Kitgum	94 (42.5%)	95 (44.0%)	189 (43.2%)
**Caretaker’s Education level**			
No school	14 (6.3%)	14 (6.5%)	28 (6.4%)
Primary school	97 (43.9%)	85 (39.4%)	182 (41.6%)
Secondary school	87 (39.4%)	96 (44.4%)	183 (41.9%)
Tertiary/ University	23 (10.4%)	21 (9.7%)	44 (10.1%)
**Weight for Age z score < −2**	26 (15.9%)	24 (15.3%)	50 (15.6%)
**Height for Age z score < −2**	60 (27.1%)	46 (21.3%)	106 (24.3%)
**Prior exposure to any pneumococcal vaccine**			
No	12 (5.4%)	8 (3.7%)	20 (4.6%)
Yes	91 (41.2%)	98 (45.4%)	189 (43.2%)
Yes, but incomplete	118 (53.4%)	110 (50.9%)	228 (52.2%)
**Prior exposure to daily oral penicillin**			
No	15 (6.8%)	8 (3.7%)	23 (5.3%)
Yes	69 (31.2%)	71 (32.9%)	140 (32.0%)
N/A (child older than 5 years)	137 (62.0%)	137 (63.4%)	274 (62.7%)
**Prior exposure to malarial chemoprevention**			
Never	22 (10%)	16 (7.4%)	38 (8.7%)
Sometimes	30 (13.5%)	28 (13%)	58 (13.3%)
Always	169 (76.5%)	172 (79.6%)	341 (78.0%)

At enrolment, 15.6% of children were underweight (weight-for-age z-score < −2), and 24.3% had stunting (height-for-age z-score < −2). Most children, 95.4%, had received at least some pneumococcal vaccination (43.2% complete, 52.2% incomplete). In Uganda, children with SCA receive SP for malaria chemoprevention each month [25]; 78.0% of participants reported always taking malaria chemoprevention before enrolment, with a further 13.3% reporting sometimes taking it (Table 1). During follow-up, mean adherence to study medication was 98.8% [4].

### Cumulative incidence of bacterial growth during follow-up among the participants by treatment arm and study site

The cumulative incidence of bacterial growth over 22 months of follow-up was 2.3% (95% CI: 1.12–4.73), Fig 1a. By the end of follow-up, the cumulative incidence was 2.6% in the DP arm and 2.1% in the SP arm (Fig 1b). By study site, the cumulative incidence reached 3.6% (95% CI: 1.4–8.7) in Kitgum and 1.2% (95% CI: 0.4–3.7) in Jinja (Fig 1c).

**Fig 1:**
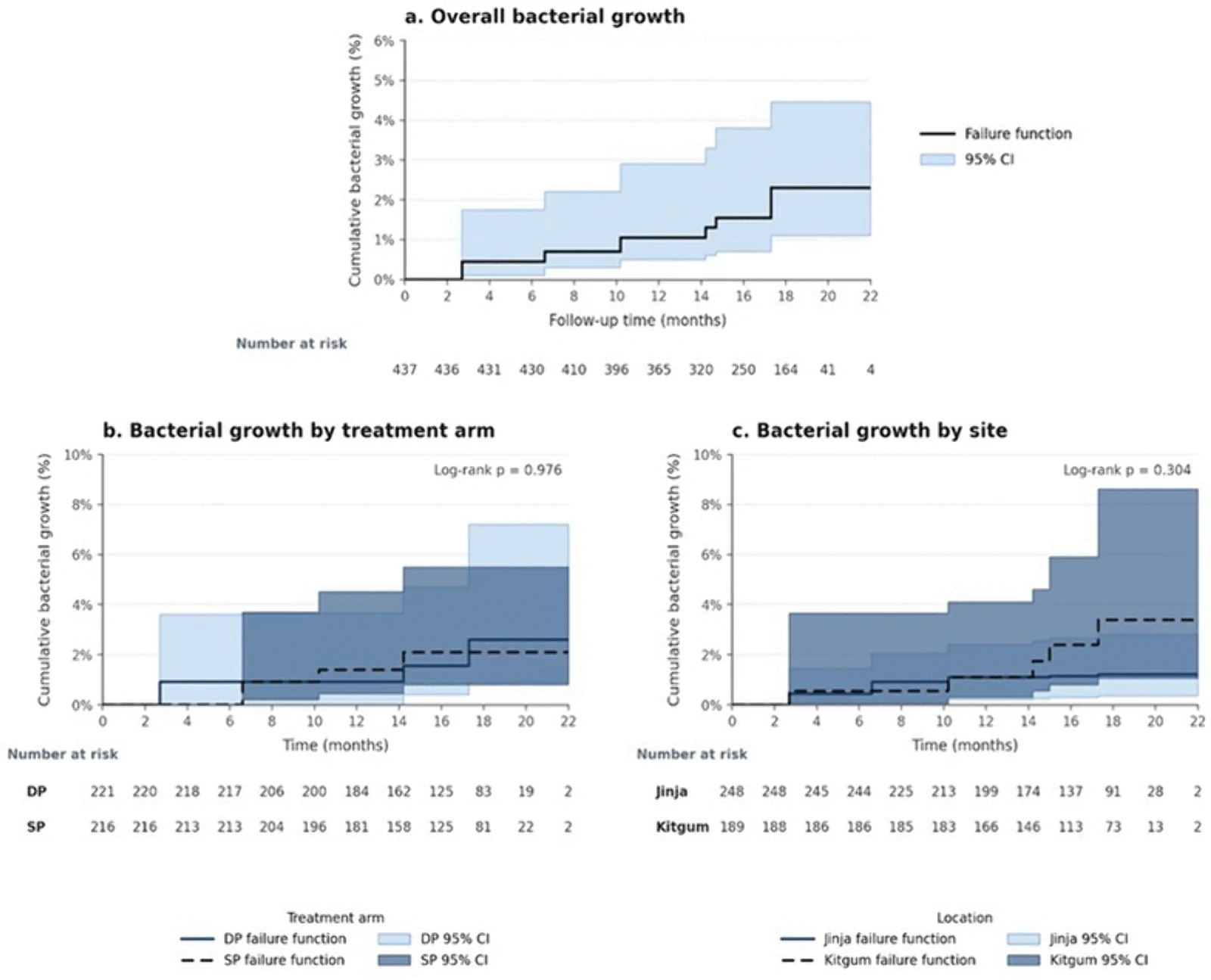
Kaplan–Meier estimates of cumulative bacterial growth among children with SCA receiving malaria chemoprevention in Uganda. a. Overall cumulative incidence of bacterial growth during the 22-month follow-up period among all study participants (N = 437). b. Cumulative incidence of bacterial growth according to malaria chemoprevention regimen: weekly DP and monthly SP. c. Cumulative incidence of bacterial growth according to study site (Jinja Regional Referral Hospital and Kitgum General Hospital).

Shaded areas represent 95% confidence intervals (CIs), and numbers at risk are shown below each panel. Bacterial growth was defined as the isolation of a bacterial pathogen from a blood culture specimen obtained during follow-up. The study was not powered to detect differences between treatment groups or study sites; therefore, these curves are presented for descriptive purposes only.

### Description of the participants who had bacterial growth during follow-up in Jinja RRH and Kitgum hospital

Eight children grew pathogenic bacteria, including three from Jinja RRH and five from Kitgum Hospital. Among these eight children, nine bacterial isolates were obtained. Four isolates were gram-positive (*Streptococcus pyogenes* [n = 1], *Staphylococcus aureus* [n = 2], and *Enterococcus faecalis* [n = 1]), while five were gram-negative organisms (*Escherichia coli* [n = 3] and *Klebsiella pneumoniae* [n = 2]). One participant (Participant 3) had two organisms identified concurrently (*Escherichia coli* and *Enterococcus faecalis*). Four of the children with isolates received DP, and the other four children received SP.

The median age at enrolment was 5 years (range 1–8), with an equal sex distribution. Based on anthropometric z-scores, two participants were stunted (height-for-age z-score <−2), and two were underweight (weight-for-age z-score <−2). At baseline, all participants had received pneumococcal vaccination (PCV-10 in Jinja and PCV-13 in Kitgum). Additionally, one six-year-old child was receiving oral penicillin prophylaxis despite not meeting current eligibility criteria, and seven of the eight children were taking SP malaria chemoprevention.

Seven of the eight children had only a single episode of bacterial growth after 1–15 months of follow-up. In addition, participants who grew pathogenic bacteria also had a higher mean number of hospitalizations (4.1, SD 2.4) vs (2.8, SD 2.5), p-value (0.063), and especially in Kitgum, Table 2.

**Table 2:** Description of the participants who had bacterial growth during follow-up in Jinja RRH and Kitgum hospital.

Participants	1	2	3	4	5	6	7	8
Site location	Jinja	Jinja	Jinja	Kitgum	Kitgum	Kitgum	Kitgum	Kitgum
Name of organism identified	*Escherichia coli*	*Klebsiella pneumoniae*	*Escherichia coli* *and* *Enterococcus faecalis*	*Staphylococcus aureus*	*Escherichia coli*	*Klebsiella pneumoniae*	*Streptococcus spp*	*Staphylococcus aureus*
Arm	DP	SP	SP	SP	DP	SP	DP	DP
Sex	Female	Female	Male	Male	Female	Male	Male	Female
Baseline clinical characteristics								
Height for Age (Z-scores) (HAZ)	−0.47	−1.57	−1.66	−0.52	−1.54	−1.18	−1.44	−0.13
Weight for Age (Z-scores) (WAZ)	−0.80	−1.34	−0.66	−0.07	−0.78	−2.43	−1.69	−0.92
Pneumococcal vaccine (type)	PCV-10*	PCV-10*	PCV-10*	PCV-13	PCV-13	PCV-13	PCV-13	PCV-13
Oral penicillin	Yes	Yes	N/A	Yes	Yes	N/A	Yes	N/A
Anti-malarial	Yes	Yes	Yes	Yes	No	Yes	Yes	Yes
During follow-up								
Number of hospitalizations	2	4	4	9	5	5	2	1
Temperature (℃)	39.5	38.5	38.9**	40	38.4	38.4	38.4	38.4
Episodes of bacterial growth during	1	2	1	1	1	1	1	1
Time to bacterial growth (months)	1	4	15.1	15	12	11.7	10.9	8
Person-years of follow-up (years)	0.94	1.53	1.46	1.24	1.21	1.18	1.15	1.65

* Incomplete Pneumococcal vaccine. ** Average temperature at the time the two isolates were identified. PCV-10: 10-valent pneumococcal conjugate vaccine; PCV-13: 13-valent pneumococcal conjugate vaccine; SP: Sulfadoxine-pyrimethamine; DP: Dihydroartemisinin-Piperaquine.

#### Antibiotic susceptibility results for the bacterial isolates.

A total of nine bacterial isolates were obtained from participants enrolled at the two study sites, including *Escherichia coli* (n = 3), *Klebsiella pneumoniae* (n = 2), *Staphylococcus aureus* (n = 2), *Enterococcus faecalis* (n = 1), and *Streptococcus agalactiae.* (n = 1). Isolates from Jinja consisted predominantly of Gram-negative organisms, while those from Kitgum included a mixture of Gram-positive and Gram-negative pathogens. Four of the children who had isolates were in the DP arm, and four in the SP arm Table 3.

**Table 3:** Antibiotic susceptibility results for the bacterial isolates.

Participants	1	2	3	3*	4	5	6	7	8
Treatment arm	DP	SP	SP	SP	SP	DP	SP	DP	DP
Site location	Jinja	Jinja	Jinja	Jinja	Kitgum	Kitgum	Kitgum	Kitgum	Kitgum
Name of organism identified	*Escherichia coli*	*Klebsiella pneumoniae*	*Escherichia coli*	*Enterococcus faecalis*	*Staphylococcus aureus*	*Escherichia coli*	*Klebsiella pneumoniae*	*Streptococcus agalactiae*	*Staphylococcus aureus*
Amikacin		I				S			
Azithromycin				R	R				S
Amoxicillin-clavulanate		I				R			
Ampicillin	R	R	R			R			
Cefazolin						R			
Cefepime	R	I	S		S	R	R		
Ceftazidime		R				R	R		
Cefotaxime	R	R			S			S	
Penicillin G			S		R		R	S	R
Vancomycin					S		S	S	
Ceftolozane-tazobactam						R			
Ceftriaxone	R	R	S		R	R	R	S	
Cefuroxime	R		S			R			
Cefoxitin		S	R		R		R		S
Ciprofloxacin	R	R		R	R	R	R		S
Doxcycline	R	I	S						
Ertapenem		S			S	S			
Gentamicin	R	R	S		R	R	R	S	S
Colistin									
Imipenem		S				S	R		
Levofloxacin		R		R	R	R	R		R
Meropenem	S	S	S		S	S	R		
Piperacillin-tazobactam		R	S			R	R		
Tigecycline						S			
Trimethoprim-sulfamethoxazole	R	R	R		S	R	R		S
Tetracycline	R	S	R		R				S
Colistin		S							
Chloramphenicol	R	S	S		S		S	R	I
Clindamycin					I		S	S	S
Erythromycin			R		R		R	R	S
Linezolid					S		S		S
Daptomycin							S		S
Tigecycline		S					S		
Oxacillin							R		
Rifampin							S		S

S: Sensitive, I: Intermediate, R: Resistant, *Second isolate for participant 3, SP: Sulfadoxine-pyrimethamine, DP: Dihydroartemisinin-Piperaquine

**Gram-negative isolates.** The *Escherichia coli* and *Klebsiella pneumoniae* isolates showed a wide range of antimicrobial resistance. Resistance was observed to ampicillin, ceftriaxone, cefepime, ciprofloxacin, levofloxacin, and trimethoprim–sulfamethoxazole. Carbapenem susceptibility was largely preserved among *Escherichia coli* isolates; however, one *Klebsiella pneumoniae* isolate had reduced susceptibility to imipenem and meropenem. Susceptibility to aminoglycosides varied, with several isolates resistant to gentamicin, whereas amikacin remained active against isolates where testing was performed Table 3.

**Gram-positive isolates.** Macrolide resistance was common, particularly among *Staphylococcus aureus*, which showed resistance to erythromycin and variable susceptibility to clindamycin. One *Staphylococcus aureus* isolate was oxacillin-resistant, consistent with a methicillin-resistant *Staphylococcus aureus* (MRSA) phenotype. Tetracycline resistance was also frequently observed among the Gram-positive isolates Table 3**.**

### Genetic markers of resistance among the bacterial isolates

Seven of the nine isolates had sufficient genomic content for the genotyping analysis and in all cases, a wide range of resistance genes were identified, Fig 2.

**Fig 2:**
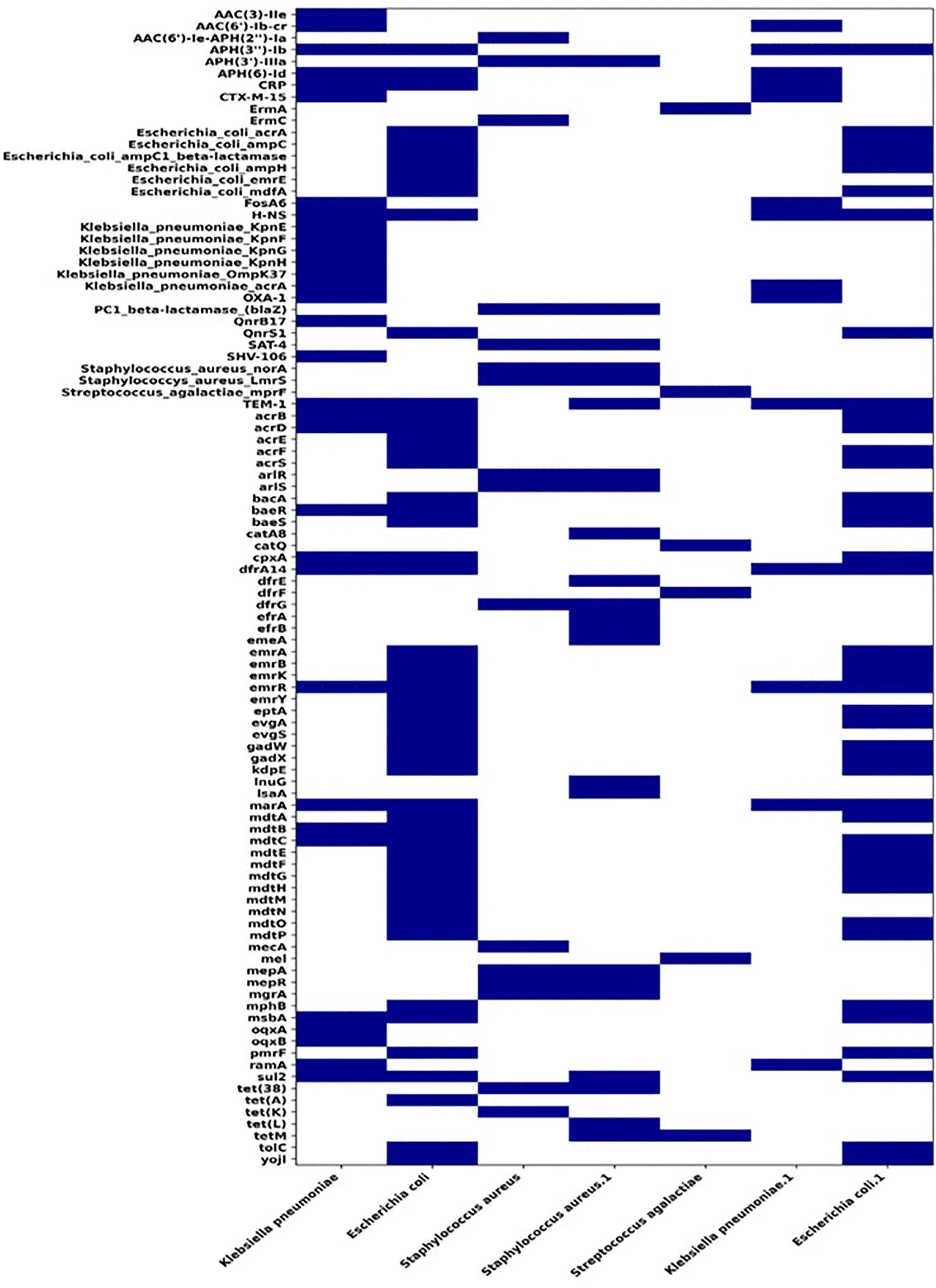
Distribution of antibacterial resistance genes across clinical bacterial isolates. ^1^: Second isolate. Blue cells indicate the presence, and white cells indicate the absence of resistance genes in each bacterial species.

The *Klebsiella pneumoniae* isolates also harbored multiple resistance determinants, including aminoglycoside-modifying enzymes (AAC (3)-IIe, AAC(6’)-Ib-cr, APH variants), β-lactamases (*bla*_OXA-1_, *bla*_SHV-106_, *bla*_TEM-1_, *bla*_CTX-M-15_), efflux pumps and regulatory genes (acrA, acrB, acrD, marA, mdtB/C, msbA, oqxA/B, ramA), and one of the isolates showed sulfonamide resistance genes (sul2), S1 Table.

The *Escherichia coli* isolates showed a broad spectrum of resistance genes, including those conferring resistance to aminoglycosides (APH(3”)-Ib), β-lactams (ampC, ampH, *bla*_TEM-1_), quinolones (qnrS1), efflux pumps and regulatory genes (acrA, acrB, acrD, mdtA-F, marA), and sulfonamides/trimethoprim (sul2, dfrA14), S1 Table.

Among the gram positive isolates, the *Staphylococcus aureus* isolates contained genes associated with resistance to aminoglycosides (APH(3’)-IIIa), β-lactams (PC1 β-lactamase blaZ), streptogramins and macrolides (SAT-4, ermA), efflux pumps (norA, mepA, mepR, mgrA), and tetracyclines (tet(38), tet(L), tetM), MecA gene (S1 Table), highlighting MRSA characteristics. The *Streptococcus agalactiae* carried genes mediating resistance to macrolides and lincosamides (ErmA), tetracycline (tetM), and trimethoprim (dfrF), consistent with both known intrinsic and acquired resistance mechanisms Fig 2.

## Discussion

This study aimed to determine the incidence and antibiotic susceptibility patterns of invasive bacterial isolates in children with SCA receiving malaria chemoprevention with weekly DP or monthly SP in Kitgum and Jinja hospitals in Uganda. We found a low cumulative incidence of invasive bacterial infections. The cumulative incidence of bacterial growth over 22 months of follow-up was 2.3% (95% CI: 1.12–4.73). During the follow-up period, the cumulative incidence of bacterial infections among children who received DP was 2.6% (95% CI: 0.9–7.2). Among children who received SP, the cumulative incidence of malaria was 2.1% (95% CI: 0.7–5.5). Notably, the few isolates identified were all multidrug-resistant. *Klebsiella pneumoniae* isolates were identified among children receiving SP, whereas *Streptococcus agalactiae* was detected only in those receiving DP. *Staphylococcus aureus* was observed in both treatment groups. The sul2 resistance gene was identified in *Escherichia coli* and *Klebsiella pneumoniae* isolates, both of which occurred in the children who received SP.

Although the observed cumulative incidence is low (2.3% (95% CI: 1.12–4.73), it is consistent with that of a recent meta-analysis of bacteremia in patients with sickle cell disease by Natasha et al., where the weighted prevalence of bacteremia in patients with SCD was 1.9% (95% CI, 1.22%−2.73%) [26]. The cumulative incidence observed in our study is lower than the 8.2% prevalence reported among children without SCA in similar settings [27,28]. The low incidence of bacterial infections may be due to the effective implementation of preventive management with vaccines and oral penicillin prophylaxis, and the small variation is likely due to the differences in the study design. However, despite these, there are still breakthrough infections among these children whose immune systems are already compromised.

The pathogen profile in our study, dominated by *Escherichia coli and Staphylococcus aureus,* is similar to the findings from a study conducted in the same location in Jinja [29]. The improvements in the preventive management of children with SCA could have changed the narrative of the presence of encapsulated bacteria such as *Streptococcus pneumoniae*. However, the predominance of *Escherichia coli and Staphylococcus aureus* in our study suggests the possibility of other underlying clinical or environmental factors influencing bacterial infection risk among children with SCA.

Eight children developed at least one bacterial infection, most commonly *Escherichia coli, Klebsiella pneumoniae, Staphylococcus aureus, Enterococcus faecalis, and Streptococcus species*. These findings underscore the persistent vulnerability of children with SCA to invasive bacterial [30] and highlight the limitations of current prevention strategies, which primarily target pneumococcal infections. Given the potential for multidrug-resistant pathogens, empiric antibiotic therapy should be selected carefully and guided by local resistance patterns whenever possible. The wide variation in time to bacterial growth further emphasizes the importance of ongoing surveillance throughout follow-up and management of children with SCA to enable early detection of infections. Early identification may help reduce morbidity, as children with documented bacterial growth experienced a higher average number of hospitalizations compared with those without identified pathogens.

The commonest phenotypic resistance was to ceftriaxone, a drug used as the standard of care in children with SCA, and to gentamycin and levofloxacin, the other most commonly used antibiotics for suspected sepsis by invasive bacteria in children with SCA in the region. This finding is similar to findings in earlier studies in Jinja RRH, where all the isolates detected were resistant to gentamycin [29]. This pattern may be due to the over-reliance on empirical treatments rather than targeted treatment guided by culture and sensitivity results, potentially contributing to selective pressure and the emergence of resistance. Empiric therapy should therefore be guided by updated local antibiogram data, with consideration of combination therapy in severely ill children and prompt de-escalation once results are available. Additionally, the presence of methicillin-resistant *Staphylococcus aureus* is of great concern as this poses a threat to the clinical management of children with SCA. Infections with MRSA are among the six leading causes of,AMR-associated mortalities globally [31].

Of great concern, a wide range of resistance genes consistent with both known intrinsic and acquired resistance mechanisms were identified, conferring multidrug resistance to multiple classes of antibiotics. The presence of the sul2 gene in this cohort of children with SCA receiving malaria chemoprevention indicates a possible cross-resistance between SP and other sulphonamides such as sulphamethoxazole. The sul2 gene encodes an alternative dihydropteroate synthase (DHPS) with reduced affinity for sulfonamides, conferring resistance to this class of antimicrobials, and is commonly found on small non-conjugative plasmids, predominantly in gram-negative bacteria [32].

Ceftriaxone is the recommended empirical therapy for suspected bacterial infections among children with SCA in Uganda [33], while sulfonamide-based therapies are recommended for malaria chemoprevention among children with SCA and pregnant women [33]. The use of sulfonamide-based therapies in malaria chemoprevention potentially predisposes bacterial pathogens to subtherapeutic exposure, driving resistance development and spread. Antibacterial resistance remains a common public concern in Uganda, as indicated in findings of the national surveillance that reported high resistance to co-trimoxazole, ranging from 55% to 70% in Escherichia coli, and 33% to 70% in Klebsiella pneumoniae [34,35]. SCA. The Sul2 findings in this cohort, although based on a small number of isolates, is consistent with this broader pattern and warrant further investigation in larger studies designed to evaluate the relationship between sulfonamide-based malaria chemoprevention and sulfonamide resistance gene carriage.

### Limitations

Although the main study was adequately powered for the primary outcome, this study was not powered to definitively distinguish an antimicrobial effect of SP. Secondly, the caretakers could have given antibiotics to the children while at home without full disclosure to the clinicians in the study. However, during the study period, the caregivers were educated and advised against self-medication.

## Conclusion

Blood cultures of Ugandan children with SCA with suspected invasive bacterial infections rarely grow pathogenic organisms. However, the children remain at risk, and the few isolates all have multidrug resistance, including to ceftriaxone, the standard-of-care intravenous antibiotic, and the alternatives of gentamicin and levofloxacin. The presence of multidrug-resistant organisms heightens the likelihood of treatment failure and severe outcomes. Additionally, the presence of sul2 indicates the possibility of sulfonamide resistance that may reduce the effectiveness of commonly used prophylactic and chemopreventive agents such as co-trimoxazole and SP, posing a challenge in low-resource settings. Sustained investments in vaccination, chemoprevention, infection prevention strategies, ongoing molecular surveillance of resistance genes, and advocacy for targeted treatment based on culture and sensitivity results are therefore critical to protect children with SCA as resistance patterns continue to evolve.

## Supporting information

S1 Table Antimicrobial resistance genes that were identified in the study isolates. (DOCX)
