# Supplementary material for: Bacterial infections and antibiotic susceptibility in children with sickle-cell anemia on malaria chemoprevention using Dihydroartemisinin-piperaquine (DP) or Sulfadoxine-pyrimethamine (SP) in Uganda
Source: PLoS One. 2026 Oct 15;21(10):e0340838. doi: 10.1371/journal.pone.0340838 (PMC13671510; doi:10.1371/journal.pone.0340838)
Supplement: Supplementary file 1 [file pone.0340838.s001.docx]

**S1 Table. Antimicrobial resistance genes that were identified in the study isolates.**

| **Mechanism group** | **Gene name** | **Gene product** |
| --- | --- | --- |
| Aminoglycoside-modifying enzymes | *aac(3)-IIe* | Aminoglycoside N-acetyltransferase |
| Aminoglycoside-modifying enzymes | *aac(6')-Ib-cr* | Aminoglycoside acetyltransferase variant |
| Aminoglycoside-modifying enzymes | *aph(3")-Ib* | Aminoglycoside phosphotransferase |
| Aminoglycoside-modifying enzymes | *aph(3')-IIIa* | Aminoglycoside phosphotransferase IIIa |
| Aminoglycoside-modifying enzymes | *aph variants* | Aminoglycoside phosphotransferases |
| β-lactamases (including ESBLs) | *ampC* | AmpC β-lactamase |
| β-lactamases (including ESBLs) | *ampH* | AmpH β-lactamase |
| β-lactamases (including ESBLs) | *blaZ* | Penicillinase |
| β-lactamases (including ESBLs) | *blaCTX-M-15* | Extended-spectrum β-lactamase (ESBL) |
| β-lactamases (including ESBLs) | *blaOXA-1* | Oxacillinase |
| β-lactamases (including ESBLs) | *blaSHV-106* | Extended-spectrum β-lactamase (ESBL) |
| β-lactamases (including ESBLs) | *blaTEM-1* | β-lactamase |
| Efflux pumps | *acrA* | Efflux pump component |
| Efflux pumps | *acrB* | Efflux transporter |
| Efflux pumps | *acrD* | Efflux transporter |
| Efflux pumps | *mdtA–F* | Multidrug transporter |
| Efflux pumps | *mdtB/C* | Efflux pump components |
| Efflux pumps | *mepA* | Efflux pump |
| Efflux pumps | *msbA* | ABC transporter |
| Efflux pumps | *norA* | Efflux pump |
| Efflux pumps | *oqxAB* | Efflux pump |
| Efflux pumps | *tet(38)* | Tetracycline efflux pump |
| Efflux pumps | *tet(L)* | Tetracycline efflux pump |
| Regulators | *marA* | Transcriptional regulator |
| Regulators | *mepR* | Regulatory protein |
| Regulators | *mgrA* | Global regulator |
| Regulators | *ramA* | Transcriptional regulator |
| Target modification/protection | *erm(A)* | Ribosomal methylase |
| Target modification/protection | *mecA* | Penicillin-binding protein 2a (PBP2a) |
| Target modification/protection | *qnrS1* | Target protection protein |
| Target modification/protection | *tet(M)* | Ribosomal protection protein |
| Target modification/protection | *dfrA14* | Dihydrofolate reductase variant |
| Target modification/protection | *dfrF* | Dihydrofolate reductase variant |
| Target modification/protection | *sul2* | Altered dihydropteroate synthase |
| Drug-modifying enzymes | *sat4* | Streptothricin acetyltransferase |

ESBL = extended-spectrum β-lactamase.
